# Assessment of the physiological effects and safety of transpulmonary chemoembolization with doxorubicin on pulmonary tissue using a human-isolated lung perfusion model

**DOI:** 10.1186/s41747-024-00532-3

**Published:** 2024-12-05

**Authors:** Alexis Slama, Hannah Steinberg, Stéphane Collaud, Özlem Okumus, Ralph-Axel Hilger, Sebastian Bauer, Hans-Ulrich Schildhaus, Clemens Aigner, Benedikt M. Schaarschmidt

**Affiliations:** 1 Department of Thoracic Surgery, Medical University of Vienna, Vienna, Austria; 2 Department of Diagnostic and Interventional Radiology and Neuroradiology, University Hospital Essen, Essen, Germany; 3 Department of Thoracic Surgery, Cologne Merheim Hospital, Witten/Herdecke University, Witten/Herdecke, Germany; 4 West German Cancer Centre, University Hospital Essen, Essen, Germany; 5 Department of Medical Oncology Essen, West German Cancer Centre, University Hospital Essen, Essen, Germany; 6 Discovery Life Sciences Biomarker Services & Institute of Pathology Nordhessen, Kassel, Germany

**Keywords:** Degradable starch microspheres, Embolization (therapeutic), Lung, Perfusion, Sarcoma

## Abstract

**Background:**

Whole lung transpulmonary chemoembolization using a combination of doxorubicin (DXO) and degradable starch microspheres (DSM-TPCE) might be a promising treatment option in soft tissue sarcoma. To pave the way for clinical studies, this study aimed to evaluate the short-term effects of DSM-TPCE with DXO using an *ex vivo* isolated lung perfusion (ILP) model.

**Methods:**

Nine lung specimens retrieved from patients undergoing lobectomy underwent *ex vivo* ILP. In groups of three, lung specimens were either treated with sole DXO, sole DSM, or combined substances (DSM + DXO). During *ex vivo* ILP, histological samples were obtained from each lung every 15 min. Quantitative DXO analysis and histopathological grading of possible tissue damage using a five-point Likert scale was performed. Two-way repeated measures ANOVA tested for differences between treatment groups and changes over time.

**Results:**

We created a preclinical *ex vivo* ILP model to simulate the effects of DSM-TPCE. In histopathological analysis, only two specimens, treated with only DXO, showed an increase in parenchymal damage over time. No significant effect of time (3.3%, *p* = 0.305) or group (23.3; *p* = 0.331) was identified. Within the lung tissue, the DXO concentration ranged from 205 to 1,244 ng/g. No significant effects could be detected regarding different treatment groups (4.9% of total variation, *p* = 0.103).

**Conclusion:**

In an *ex vivo* ILP model using human lung lobes, the physiological effects of DSM-TPCE with DXO could be tested. Neither increased DXO concentrations in lung tissue nor histopathological changes indicating early lung toxicity were observed.

**Relevance statement:**

An *ex vivo* ILP model using human lung specimens did not show any signs of early lung toxicity after transpulmonary chemoembolization with DXO. These results support further evaluation of DSM-TPCE in phase I/II trials.

**Key Points:**

- Transpulmonary chemoembolization can be investigated in an *ex vivo* ILP model.
- DSM did not increase DXO in normal lung tissue.
- DSM did not increase parenchymal toxicity compared to the control groups.

**Graphical Abstract:**

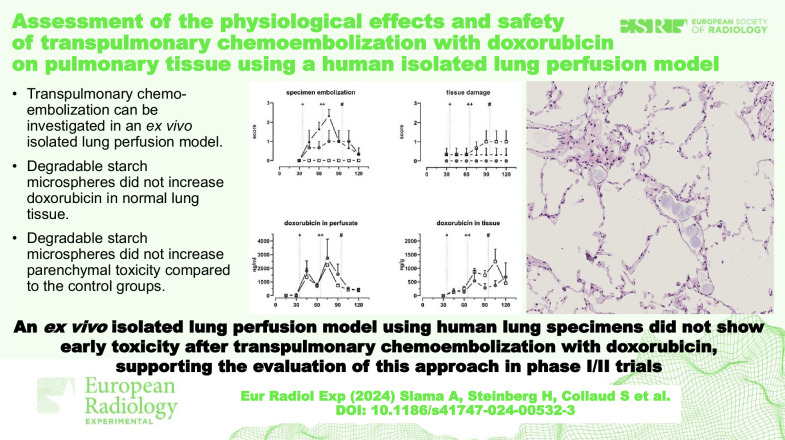

## Background

Soft tissue sarcoma (STS) is a tumor with a rising incidence to an estimated number of 13,400 new cases and 5,140 estimated deaths in the United States in 2023 [1]. Despite advances in locoregional tumor treatment, up to 32% of STS patients develop distant metastases throughout their disease [2]. In more than 50% of STS patients, the lung is the only affected organ [3]. If complete metastasis removal is possible, pulmonary metastasectomy is deemed beneficial, with 5-year survival rates of up to 52% [4]. However, this treatment is only possible in oligometastatic patients. In patients with multilocular metastatic spread, systemic chemotherapy with doxorubicin (DXO), whose efficacy is limited because of toxicity-related dose restrictions, is the only treatment option at the moment [5, 6]. Here, the localized application of DXO could allow for the delivery of higher doses to the pulmonary metastases and reduce systemic toxicity.

Such concepts were first employed in liver tumor treatment [7]. Cancerous tissue in the liver is highly dependent on the arterial blood supply, unlike normal liver parenchyma supplied by the portal venous system. This has led to the development of various intraarterial treatments for hepatic malignancies [8–10]. However, the considerable anatomical and physiological differences between the lungs and the liver complicate translating such concepts to the treatment of pulmonary metastases.

While blood supply via the bronchial artery can be observed in lung metastases from colorectal cancer as recently described by Boas et al in a phase I study on pulmonary chemoembolization [11], the literature shows that especially in sarcoma, blood supply relies heavily on the pulmonary artery. Nagashima et al observed that sarcoma metastases reach the lung via the pulmonary artery and have a predominant supply via the pulmonary artery [12]. These initial findings were verified by Milne et al, who could demonstrate *in vitro* and *in vivo*, that 48% of metastases were entirely and 36% were predominantly supplied via the pulmonary artery [13].

Considering the limited role of the bronchial arteries arising from the systemic circulation, the initial suggested approach to an unselective treatment of STS lung metastases was *in vivo* isolated lung perfusion (ILP). Here, the pulmonary arteries are accessed surgically (either by thoracotomy or transsternal incision) and sequentially clamped out from the pulmonary circulation. Then, the pulmonary arteries and veins of the isolated lung are flushed with a chemotherapeutic agent. Subsequently, metastases are resected in most cases [14, 15]. Considering this treatment’s complexity and invasiveness, *in vivo* ILP has not been widely adopted. Also, no repeated applications in a single patient have been reported. To overcome such limitations, selective catheterization of the pulmonary arteries was tested [16].

Adding degradable starch microspheres (DSM) to the chemotherapeutic agent seems useful in improving treatment efficacy. In a rat model, the addition of DSM led to a 60-fold increase of the chemotherapeutic agent in metastatic tissue compared to the intravenous concentration [6]. However, only mild inflammatory changes could be found within three days of treatment, and no residual changes were observed 6 months after transpulmonary chemoembolization (TPCE) with DSM in a porcine model [17, 18]. Initial experiences with selective TPCE in human patients with various metastases were promising [11, 19, 20].

In a phase I ILP study using DXO as a chemotherapeutic agent, Burt et al reported a considerate decrease of pulmonary function in the majority of sarcoma patients administered 40 mg/m^2^ and the destruction of the treated lung in one patient receiving 80 mg/m^2^ [14]. To ensure patient safety and avoid pulmonary toxicity, it is necessary to study the complex interplay of DSM and DXO in a controlled setting before clinical use in STS patients.

Therefore, this study aimed to investigate the effects of TPCE with DSM and DXO on human lung parenchyma in a previously established *ex vivo* ILP model [21, 22].

## Methods

### Patients

Patients necessitating pulmonary lobectomy were eligible to participate in this study. A multidisciplinary tumor board decided upon the indication for surgical treatment. The study was approved by the local ethics committee of the University of Duisburg-Essen (date of approval January 10, 2018; identifier 17-7802-BO). Written informed consent was obtained from the patients before surgery.

### TPCE

**Fig. 1:**
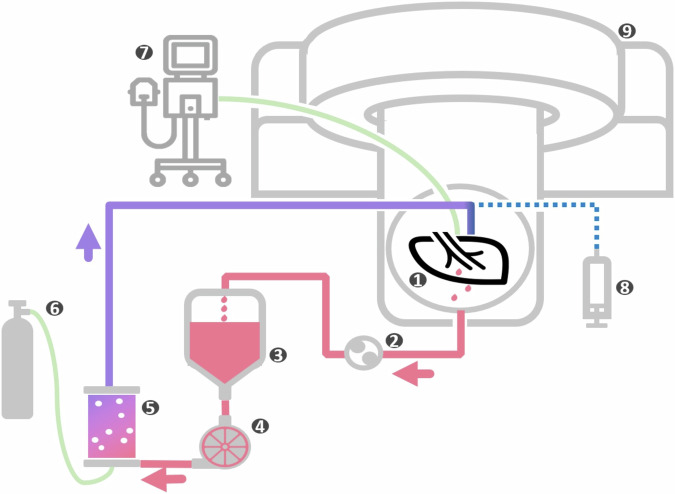
Schematic of the perfusion circuit. The diagram illustrates the setup used for lung perfusion, including key components: (1) the perfused lung lobes, (2) the roller pump, (3) the reservoir, (4) the pump, (5) the oxygenator, (6) the gas supply, (7) ventilator, (8) automatic syringe pump with treatment drug, and (9) CT scanner

**Fig. 2:**
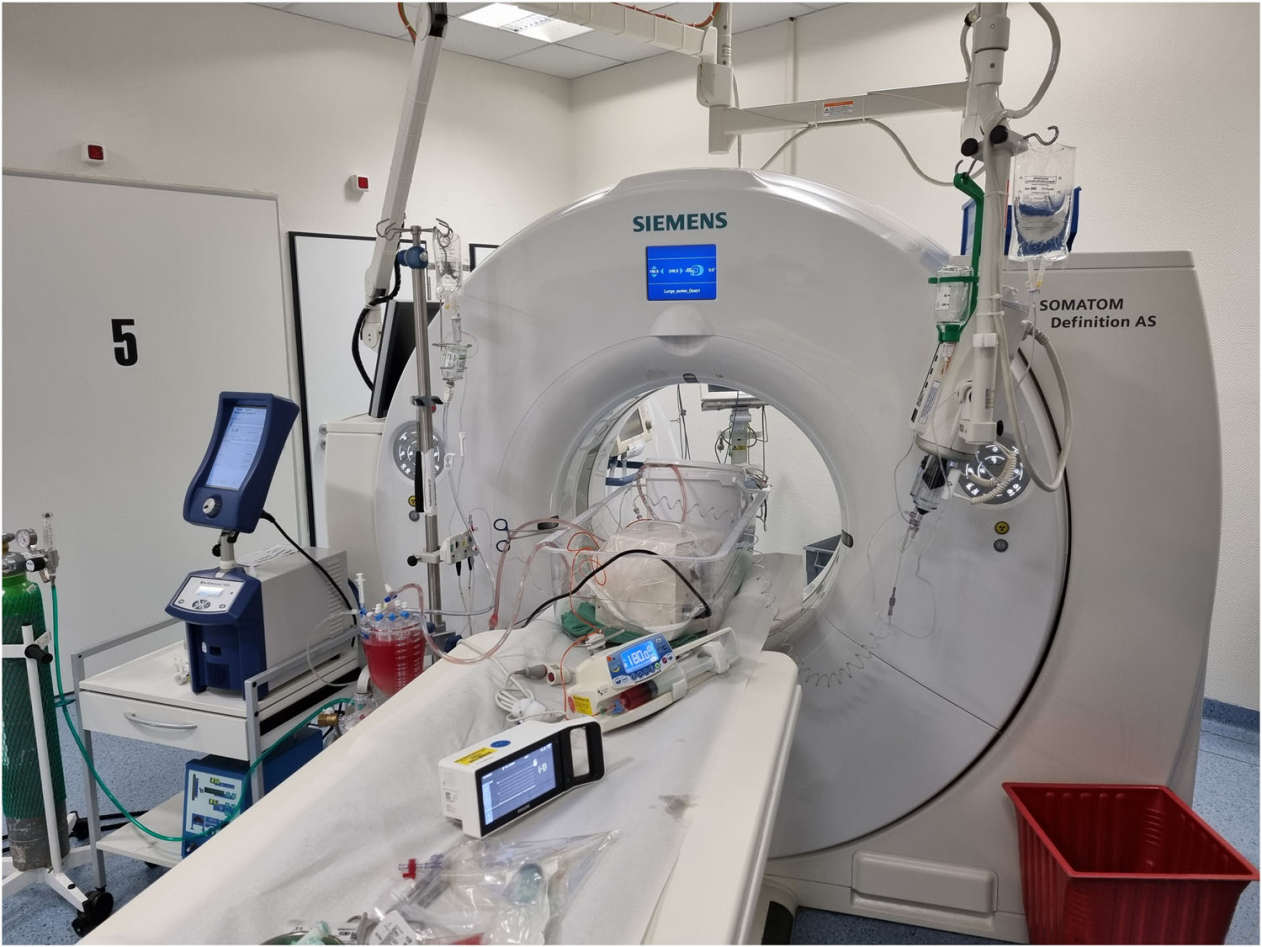
Actual experimental setup

Specimen preparation and ILP were performed similarly to our previous series [22]. In all cases, the necessary preparations were performed by an experienced, board-certified thoracic surgeon. Immediately after lobectomy and removal of the specimen, it was flushed with 1 L of cold (4 °C) preservation solution (Perfadex Plus®, XVIVO, Göteborg, Sweden) supplemented with 5,000 IU of unfractionated heparin. Silicone cannulas were sutured to the arterial vascular and bronchial stumps using uninterrupted running sutures (4/0 Prolene®, Johnson & Johnson Medical GmbH, Norderstedt, Germany). Previously separated vascular and bronchial branches of the superior segment (S6) were reanastomosed (side-to-side) to the intermediate branches when needed. After complete inflation with an Ambu® bag, the lung lobes were kept cold (4 °C) until initiation of ILP, which was carried out with a modified extracorporeal membrane oxygenation—ECMO circuit in a 64-slice computed tomography (CT) scanner (SOMATOM Definition AS, Siemens Healthineers, Forchheim, Germany). The circuit was primed with 1,220 mL of hyper-oncotic acellular colloidal perfusate (32.8 g/L succinate gelatine; 32.8 g/L human albumin; 6.6 g/L glucose). After a protective warming-up period of 20 min, lungs were ventilated (Dräger, Evita XL, Lübeck, Germany) in a protective manner with tidal volumes based on the patient’s preoperative pulmonary function test (8 beats per min, a 1/2 inspiratory/expiratory ratio, 0.4 fractions of inspired oxygen, and 5–10 cmH_2_O of positive end-expiratory pressure). Perfusion flow was set at 40% of the predicted cardiac output through those lobes. Dynamic lung compliance was calculated according to tidal volume/(peak airway pressure/positive end-expiratory pressure) (Figs. 1 and 2).

One study participant performed the drug preparation to keep the rest of the team blinded to the administered substance. In random order, three different formulations were prepared for the experiment:

- DXO: Doxorubicin 20 mg (10 mL), 22.5 mL Ultravist® 300, Bayer Vital GmbH, Leverkusen, Germany);
- DXO + DSM: Doxorubicin 20 mg (10 mL), 7.5 mL Embocept S (PharmaCept GmbH, Berlin, Germany), 15 mL Ultravist® 300, (Bayer Vital GmbH, Leverkusen, Germany);
- DSM: 7.5 mL Embocept S (PharmaCept GmbH, Berlin, Germany), 25 mL Ultravist® 300 (Bayer Vital GmbH, Leverkusen, Germany).

After reaching normothermia (35 °C) and ventilation of the lobes, a baseline assessment was carried out (t30). The following functional variables were recorded: pulmonary artery pressure (PAP), airway pressure, pulmonary vascular resistance (PVR), and a blood gas analysis. CT scans of the lung followed by perfusion imaging for 60 s were performed. Ten seconds after the start of data acquisition, 15 mL of contrast media (Ultravist® 300, Bayer Vital GmbH, Leverkusen, Germany) followed by a saline flush of 15 mL were injected with an automated syringe infusion pump (2 mL/min, Injectomat® MC Agila, Fresenius Kabi, Bad Homburg vor der Höhe, Germany).

**Fig. 3:**
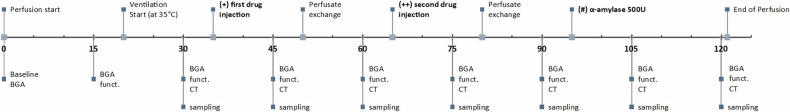
Timeline of the experimental protocol. The horizontal scale represents time in minutes. Interventions are marked above the axis, including perfusion start, ventilation initiation, drug administration, and perfusate exchange. Data recordings and tissue sampling are reported below the axis. BGA, Blood gas analysis of the perfusate; funct., Recording of functional data; DSM, Administration of degradable starch microspheres; DXO, Doxorubicin; CA, Contrast agent

One perfusate sample and two histopathological samples with a total size of 1–2 cm³ were obtained. One histopathological sample was stored in 4% neutral buffered formalin for further analysis, and the other samples were cooled immediately to quantify the contained chemotherapeutic agent later. Data and sample acquisition were repeated every 15 min (t45, t60, t75, t90, t105, and t120). After the initial analysis at t30, 1 mL of the prepared drug was preserved for further analysis. Fifteen milliliters of the prepared drug was administered using an automated injector. After data and sample acquisition at t45, the perfusate was fully exchanged to remove the investigated substances as much as possible and to counteract arterial recirculation. After a third data acquisition at t60, the remaining 15 mL of the prepared drug was administered as a second treatment cycle, followed by another perfusate exchange and data point. Ultimately, alpha-amylase (Merck KGaA, Darmstadt, Germany) was administered via an arterial access route to induce DSM hydrolysis. The experiment was terminated after two additional data points at t105 and t120 (Fig. 3).

### Histopathological analysis

As previously described [22], samples were embedded and sectioned with a 3–4 μm thickness and stained with hematoxylin and eosin. One pathologist blinded to the experiments analyzed the extent of vascular embolization as follows: 0 = no particles; 1 = single particles, no aggregates; 2 = single particles, few aggregates (< 50% of all particles consisting of aggregates); 3 = at least 50% of particles appeared as large aggregates. Pulmonary damage was assessed using a modified 5-point ordinal scale initially proposed by Nishina et al [23] using a combined analysis of alveolar congestion, hemorrhage, infiltration or aggregation of neutrophils in the airspace or vessel wall, and thickness of alveolar wall/hyaline membrane formation and endothelial damage (0 = no damage; l = mild damage; 2 = moderate damage; 3 = severe damage; and 4 = maximum damage).

### DXO concentration analysis

The concentrations of DXO in the samples were determined as described previously [24]. In brief, each liquid sample was taken into a sodium-ethylenediaminetetraacetic acid tube and centrifuged. DXO was bound to silica gel. After centrifugation, the upper phase was separated from the silica-bound DXO fraction (lower phase). The fractions were extracted with acidified methanol.

Tissue samples were efficiently homogenized using a laboratory disperser (Ika-Ultra-Turrax, IKA-Werke GmbH & Co. KG, Staufen, Germany).

Samples were deprotonated by adding ice-cold methanol/acetonitrile solution and stored for at least 2 h at -20 °C. After centrifugation, the upper phase was separated.

For the quantification of DXO, volumes of 200 µL of each fraction were injected into the following validated reversed-phase high-performance liquid chromatography—RP-HPLC system: a Waters 2695 Separation Module connected to a Waters 474 Scanning Fluorescence Detector (excitation wavelength: 470 nm, emission wavelength: 550 nm); solvent system: 1 mL/min 28% acetonitrile and 72% aqua (20 mM di-potassium hydrogen phosphate, 20 mM 1-heptanesulfonic acid sodium salt, pH = 7.50 with orthophosphoric acid) at 0 min to 40% acetonitrile and 60% aqua within 30 min at room temperature (RT); column: Waters Symmetry, 250 × 4.6 mm; software: Waters Empower2 Chromatography Manager. The assay’s detection limit is 0.1 ng/mL for DXO. The intra-assay coefficient of variation for DXO is given to 4.3%, and the inter-assay coefficient of variation to 4.9%.

### CT perfusion imaging analysis

The Syngo.CT Dynamic Angio workflow of the syngo.via software (VB40, Siemens Healthineers, Forchheim, Germany) was used to analyze images. For each patient, the two radiologists, drew a central region of interest in the pulmonary artery in consensus for each image acquisition at t30, t45, t60, t75, t90, t105, and t120 (H.S. and B.M.S.). Then, nonoverlapping regions of interest were placed in the lung specimen’s upper, mid, and lower peripheries, with sizes ranging from 3 cm^2^ to 5 cm^2^ appropriate to the lobe size. The time to peak (TTP) of the regions of interest in the pulmonary artery was subtracted from the mean TTP value of all three peripheral regions of interest for each patient and each timepoint to account for variations due to the length of the vascular anastomoses and alterations caused by consolidation or dystelectasis of the parenchyma.

### Statistical analysis

GraphPad Prism 10.0 (GraphPad Software, San Diego, CA, USA) was used for data analysis and plotting. Two-way repeated measures ANOVA (main effect only model) were used. The analyses were corrected for the lack of sphericity (Geissner-Greenhouse) and multiple comparisons (Dunnett). This approach allowed us to compare variables over time and according to group assignment. In analyses regarding drug concentration, only the respective groups where the pharmaceutical agent was used were included in the ANOVA. ANOVAs were reported as F (degrees of freedom) or as a percentage of total variance followed by a *p*-value for each effect (group and time). Values of *p* lower than 0.050 were considered statistically significant.

## Results

### Cases

All but one patient had a verified lung malignancy in stage I/II. Median age was 68 years (range 32–79). The median total lung capacity (TLCr) of the patients was 5.49 L (range 4.05–9.32), and their median diffusing capacity for carbon monoxide was 4.89 L (2.78–11.62). Three patients had a normal body mass index (< 25), with all others overweight and obese (*n* = 6). Median body mass index was 26.7 (19.0–42.0). All patients underwent lower lobe lobectomy (right or left; *n* = 7) or right-sided lower bilobectomy (*n* = 2). The surgical access was either a thoracotomy (*n* = 4) or a uniportal video-assisted thoracoscopy (VATS; *n* = 5). Depending on the resected lobes, the number of lung segments varied between 4 (in the left lower lobes) and 7 (in the right combined middle and lower lobe specimens, Table 1).

**Table 1:** Patient demographics

Group	DSM + DXO			DSM			DXO		
Age	64	32	69	79	47	68	72	65	76
Sex	Male	Male	Female	Female	Female	Female	Female	Male	Female
BMI	30.6	24.7	26.7	19	18.4	30.5	25	31.4	42.1
TLCr, (L)	9.32	8.69	5.66	4.05	5.49	4.53	4.88	6.76	5.011
TLCr, (% of predicted)	120	106	114	81	108	92	110	101	108
DLCO, (L)	4.48	11.61	3.43	NA	5.31	4.24	2.78	5.31	5.3
Estimated CO, (L)	6.32	5.9	4.84	4.12	4.09	5.12	4.34	5.69	5.8
Surgical access	Thoracotomy	VATS	VATS	Thoracotomy	Thoracotomy	VATS	VATS	Thoracotomy	VATS
Lobe	RLL	LLL	LLL	RML + RLL	RLL	RLL	RLL	RML + RLL	RLL
Perfused segments	5	4	4	7	5	5	5	7	5
Histopathology	NSCLC (SCC)	Carcinoid	NSCLC (AC)	NSCLC (AC)	Abscess	NSCLC	NSCLC	NSCLC (AC)	Carcinoid

*AC* Adenocarcinoma, *BMI* Body mass index, *DLCO* Diffusing capacity for carbon monoxide*, DSM* Degradable starch microspheres, *DXO* Doxorubicin, *L* liter, *LLL* Left lung lobe, *NA* Not available, *NSCLC* Non-small cell lung cancer, *RLL* Right lung lobe, *RML* Right middle lobe, *SCC* Squamous cell carcinoma, *TLCr* Total lung capacity, *VATS* Video-assisted thoracoscopic surgery

### Perfusion dynamics

**Fig. 4:**
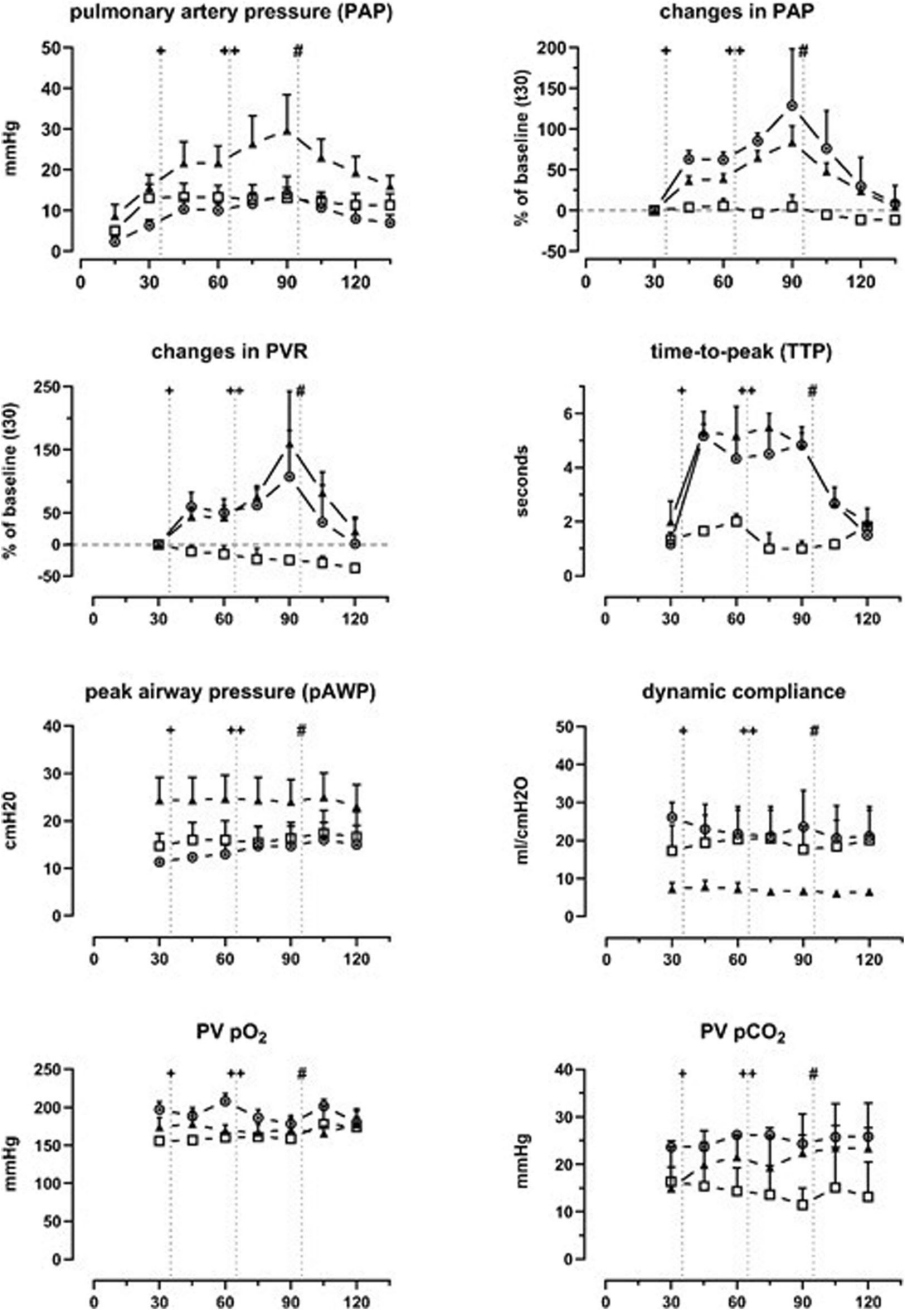
Recorded parameters across the three experimental groups: circle ◉ = DSM + DXO; triangle ▲ = DSM; square  = DXO. Each panel represents the changes over time for one variable. Data is presented as group means with error bars indicating the upper standard error of means. The measurements were recorded at specific time points. The vertical lines indicate the time points where a drug was administered. ^+^First treatment, ^++^Second treatment, ^#^Alpha-amylase. DSM, Degradable starch microspheres; DXO, Doxorubicin; PVR, Pulmonary vascular resistance; PV, Pulmonary vein; pO_2_/pCO_2_, Partial pressure of O_2_ and CO_2_ in the perfusate

In both groups, DSM, PAP, and PVR increased significantly compared to the baseline. After the first DSM administration, the mean ± standard deviation PAP increased by 62.0 ± 15.8% (DSM + DXO) and 39.0 ± 9.9% (DSM) compared to the baseline. After the second administration, the increase reached 128 ± 120% and 83 ± 34%. Computed PVR increased accordingly in both groups by 107 ± 127% and 160 ± 143%. Accordingly, average TTP in CTPI increased significantly in embolized specimens (DSM + DXO, 1.2 ± 0.8 s at t30 to 4.8 ± 1.2 s at t90; DSM, 2.0 ± 1.3 s to 4.8 ± 0.8 s; and DXO, 1.3 ± 0.3 s to 1.0 ± 0.5 s). In the DXO controls, no significant changes were found regarding PAP (*p* = 0.440), whereas PVR showed a linear decrease over time (*p* < 0.001). Consistently, TTP showed no significant changes over time (*p* = 0.780). After administration of alpha-amylase (500 IU) at t95, PAP, PVR, and TTP values in embolized lobes reverted to values comparable with the baseline: PAP, DSM + DXO + 8.3%, DSM + 4.0%; PVR, DSM + DXO + 1.1%, DSM + 20.8%, TTP, DSM + DXO 1.5 ± 0.5 s, DSM 2 ± 0.9 s (Fig. 4).

### Ventilation and gas exchange

Two-way ANOVAs showed no significant differences in airway pressure (over time F(1.502, 12.01) = 1.156, *p* = 0.331; between groups F(2, 6) = 2.046, *p* = 0.210), lung compliance (over time F(1.837, 14.70) = 0.252, *p* = 0.763; between groups F(2, 6) = 2.266, *p* = 0.185), and gas concentrations (pO_2_: time F(3.032, 24.25) = 0.8657, *p* = 0.473; groups F(2, 6) = 1.904, *p* = 0.229; pCO_2_: time F(2.285, 18.28) = 0.6243, *p* = 0.567; groups F(2, 6) = 1.059, *p* = 0.404). Including all cases, the mean peak AWP was 16.8 ± 6.7 cmH_2_O at baseline and 18.2 ± 4.2 cmH_2_O after 120 min (slope 95% confidence interval -0.052 to 0.093; *p* = 0.565). Dynamic lung compliance was 16.9 ± 9.4 mL/cmH_2_0 at baseline and 15.9 ± 8.1 mL/cmH_2_O after 120 min (slope 95% confidence interval -0.11 to 0.08; *p* = 0.735). pO_2_ of the perfusate was 175.6 ± 20.7 mmHg at baseline and 177.3 ± 2.8 mmHg at 120 min (slope 95% confidence interval -0.20 to 0.25; *p* = 0.817, Fig. 3).

### Metabolism

**Fig. 5:**
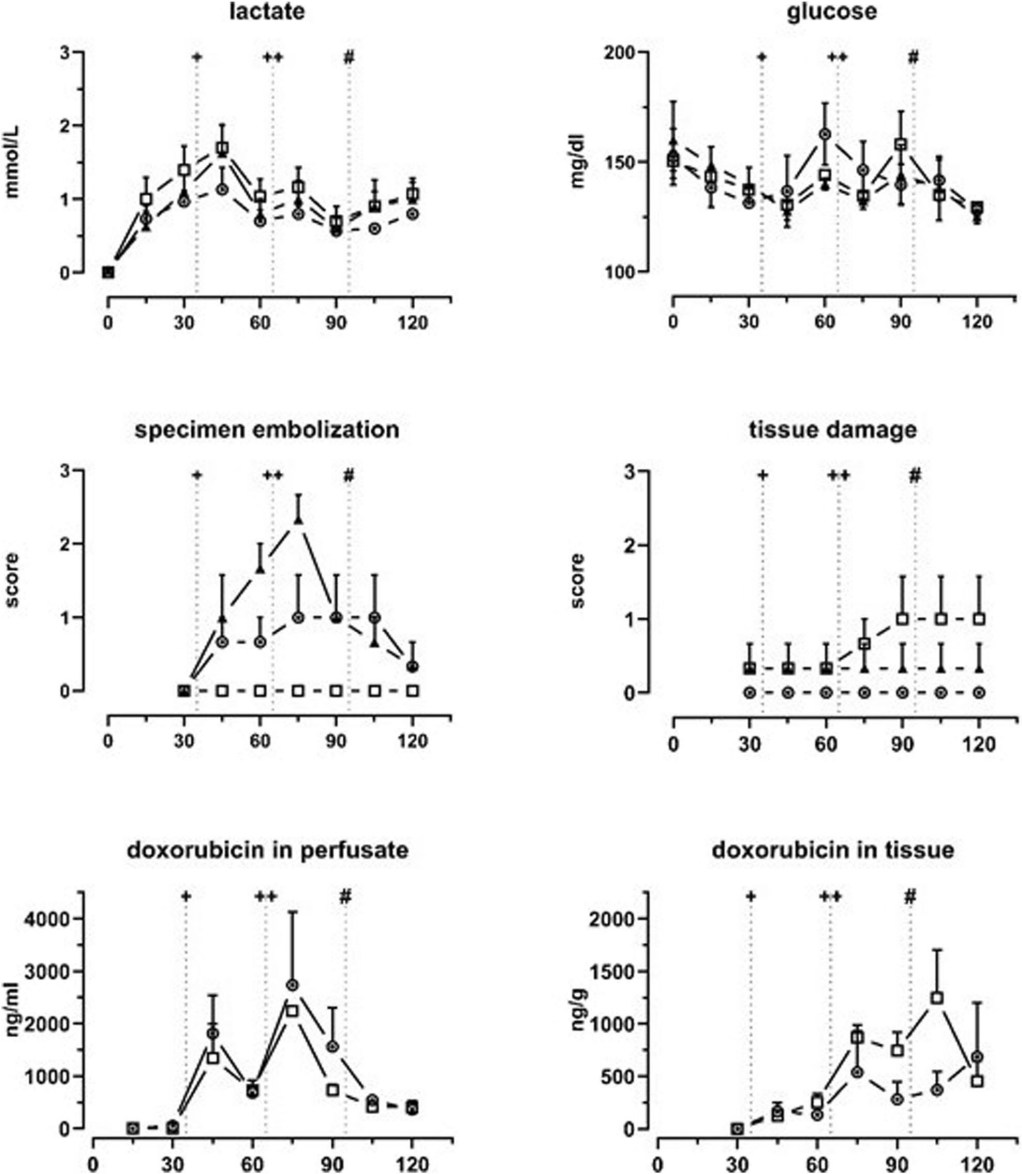
Variables from perfusate and tissue samples across the experimental groups: circle ◉ = DSM + DXO; triangle ▲ = DSM; square  = DXO. Each panel represents the changes over time for one variable. Data is presented as group means with error bars indicating the upper standard error of the means. The upper panels show lactate and glucose concentrations over time for each group. The middle panels present the mean scores assigned by the pathologist. The lower panels depict the concentration of DXO in the perfusate and the tissue in the DSM + DXO ◉ and DXO  groups. ^+^First treatment, ^++^Second treatment, ^#^Alpha-amylase. DSM, Degradable starch microspheres; DXO, Doxorubicin; PVR, Pulmonary vascular resistance; PV, Pulmonary vein; pO2/pCo2, Partial pressure of O_2_ and CO_2_ in the perfusate

Perfusate was exchanged twice throughout the experiments (t50, t80) to reduce the circulating DSM and DXO in the system. This also increased glucose and reduced lactic acid at the subsequent measurements (t60, t90). Two-way ANOVA showed variation over time (glucose, F(2.761, 19.33) = 3.012, *p* = 0.059; lactic acid, F(2.801, 22.41) = 34.03, *p* < 0.0001) and no variation between groups (glucose, F(2, 5) = 0.06268, *p* = 0.940, lactic acid F(2, 6) = 0.5708, *p* = 0.593) (Fig. 5).

### Histopathological findings and drug concentration

Repeated DSM administration in both treatment groups increased embolization in the capillaries in HE staining. Embolization grades (0–3) increased until t75. ANOVA showed no significant variation over time (35.7%; *p* = 0.080) but between both groups (3.91%; *p* = 0.002). After adding alpha-amylase, mean scores reverted to 0 or 1 at the end of the experiment.

**Fig. 6:**
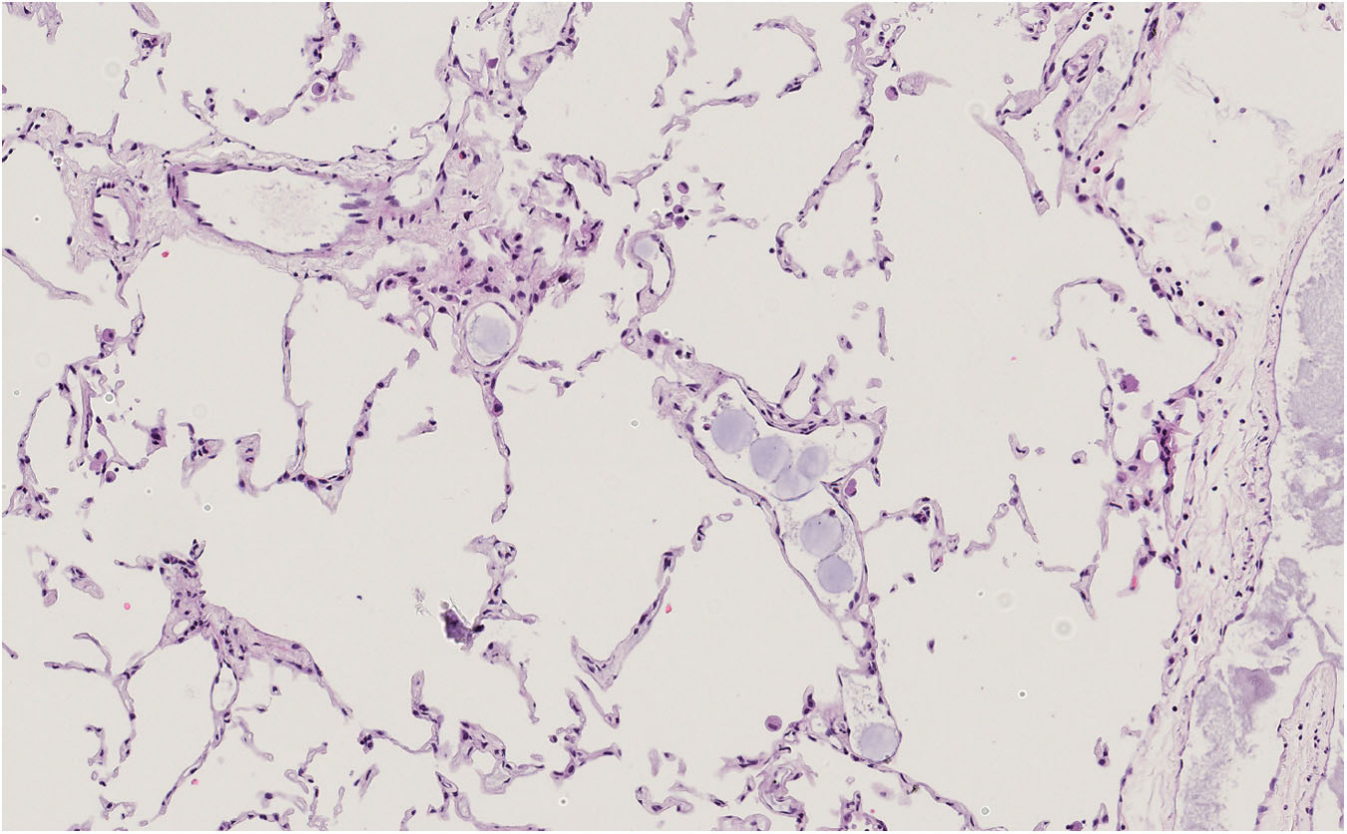
Histological section of a lung specimen after the second infusion of DSM and DXO (t75). Single DSM particles and few aggregates can be found, equaling a score of 2. No early changes indicating lung toxicity can be found. DSM, Degradable starch microspheres

Only three specimens presented a score > 0 regarding the score assigned to quantify parenchymal damage. One was in the DSM group, and two were in the DXO group (Fig. 6). In the one specimen in the DSM group, however, mild tissue damage could already be observed at t30 and no increase in tissue damage could be observed over time. No significant effect of time (3.3%, *p* = 0.305) or group (23.3%; *p* = 0.331) was identified.

In both groups with DXO, drug concentration in the perfusate fluctuated per drug administration and perfusate exchanges (t50, t80), with time showing a significant effect (*p* = 0.018). The concentration range after the second administration (t65) was 2,115–5,173 ng/mL, and no difference was seen between both groups (*p* = 0.296). Within the tissue, concentration showed less fluctuation. After the second administration, the concentration ranged from 205–1,244 ng/g. No significant effects on tissue concentration could be detected (time, 34% of total variation, *p* = 0.056; group, 4.9% of total variation, *p* = 0.103) (Fig. 5).

## Discussion

New evidence in the field of TPCE illustrates the use of this new procedure as a palliative treatment option in patients with pulmonary metastases. Prior to its use in the treatment of pulmonary metastases in STS patients, further insights concerning the toxicity of this new procedure are needed. This study investigating the impact of unselective DSM-TPCE combined with DXO provided the following four key findings. First, we succeeded in creating a preclinical *ex vivo* ILP model to evaluate the effects of DSM-TPCE on human lungs. Here, we successfully assessed treatment effects, reproducing physiological changes caused by this procedure in previous preclinical models. Second, we confirmed the reversal of these effects after adding alpha-amylase into the circulating perfusate. Third, we found no significant differences regarding the DXO concentration in normal lung tissue between the DXO and the DXO + DSM group. Fourth, we did not observe early parenchymal damage indicating early lung toxicity in the DXO + DSM group.

Especially in treating lung metastases in STS patients, localized tumor treatment of the lung harbors great potential. *In vivo* ILP was initially proposed to treat diffuse metastases ineligible for surgery or before resection. However, the invasiveness of ILP and the possibility of accompanying complications thwarted general clinical use apart from selected centers [14, 15]. Here, DSM-TPCE might be a possible treatment option, which has already been proposed in initial studies for various types of pulmonary metastases. Although preclinical evidence with different animal models is available, further research is necessary to evaluate the impact of this procedure on human lung tissue. This is particularly important to avoid lung toxicity by DXO, which is still considered a cornerstone in STS treatment. In this regard, our institution’s *ex vivo* ILP model might provide a unique opportunity. Using resected lung lobes, this model facilitates the investigation of the effects of various drugs or treatment forms on human lung tissue in the safe environment of an *ex vivo* model [21, 22, 25]. The physiological impact of DSM-TPCE was comparable to the previously reported effects in our *ex vivo* ILP model and previous animal experiments [18, 22]. As expected, we observed a steady increase of PAP, TTP, and the histopathological extent of embolization in the DSM and DSM + DOX, which could be reversed after alpha-amylase administration.

These findings are in line with prior studies. In a rat model, Schneider et al began to notice a reestablishment of a slow blood flow 6–12 min after embolization with DSM particles and a complete physiological flow after 12–26 min [26]. In pigs, Pohlen et al observed a “complete normalization of pulmonary arterial blood flow on DSA” in all animals, and Barabasch et al found a small number of foreign body granulomas in only one-third of the examined pigs [17, 18].

The analysis of the DXO concentration demonstrated comparable concentrations in the perfusate for the DOX and the DSM&DOX group. The exchange of the perfusate at t50 and t80 led to the desired effect, as we observed a statistically significant decrease in the DXO concentration in the perfusate.

However, the DXO washout by perfusate exchange was less pronounced in the lung tissue. This was most likely due to the accumulation of DXO in the extravascular compartment, which led to no significant changes over time.

Unexpectedly, the addition of combined administration of DSM and DXO (DSM + DXO) did not lead to an increased DXO concentration in the lung tissue compared to the sole infusion of DXO. These findings differ from the results reported in the literature. In a rat model, Pohlen et al described a two-fold increase of DXO concentrations after chemoembolization with DSM particles compared to sole DXO infusion. The use of a different amilomer can potentially explain this difference. While Pohlen et al used DSM particles with a mean diameter of 45 ± 7 μm (amilomer 25/45), we used DSM particles with an average sphere diameter of 50 µm (range, 20–200 µm) [27]. As DSM particles tend to aggregate in vessels, it is possible that small vessels supplying normal lung tissue were occluded by aggregates of these slightly larger DSM particles, leading to lower concentrations of DXO in the normal lung tissue.

The results of the histopathological analysis reflect these findings. Here, we saw only minor harm on the lung tissue over time in selected cases (*n* = 0 in the DSM group, *n* = 2 in the DXO group, *n* = 0 in the DSM + DXO group) and could not detect statistically significant differences regarding short-term lung toxicity. These results reflect the experiences of various animal studies. In DSM-TPCE with carboplatin, Schneider et al observed no changes in the thickness of alveolar septae or capillary permeability as early signs of pulmonary toxicity [26]. Similar findings were made by Pohlen et al after performing DSM-TPCE with carboplatin in a pig model with no parenchymal alterations detectable in histopathological analysis six months after treatment [17]. For DSM-TPCE with DXO, Barabasch et al reported only mild inflammatory changes in a pig model after up to 72 h [18]. Hence, our experiment provides further arguments for the safety of DSM-TPCE with DXO.

Our study has some limitations. While some limitations are associated with the *ex vivo* ILP model and have been discussed in full in our previous papers, some demand further discussion. First, we decided not to perform histopathological and DXO analysis in tumorous tissue. This decision was based on the different characteristics of the tumors in the lung specimens used for *ex vivo* ILP and STS. As the main surgical treatment form for STS metastases is metastasectomy by tissue-sparing wedge resections, resected lung lobes with STS metastases are not available. To further advance the current knowledge about treatment efficacy mainly based on animal models, further evaluation should be conducted in phase I/II studies. Therefore, our study focused on the potential toxicity of this procedure for normal lung tissue to ensure the safety of participants in these trials. Second, the sample size is small. Like in our prior study, the main goal of the present analysis was to demonstrate the feasibility of simulating DSM-TPCE in an *ex vivo* ILP model and the possibility of analyzing parenchymal damage, as well as concentrations of chemotherapeutic agents in the treated specimen. Third, we did not include a control group receiving any therapeutic component in our trial design as the safety of bland embolization using DSM was demonstrated already in our prior work [22]. Fourth, there might be increased exposure of the lung specimen to DXO compared to an *in vivo* administration due to the less than physiologic flow and the lack of continuous DXO clearance. To circumvent this restriction as best as possible, we decided to exchange the perfusate regularly and thus reduce the amount of recirculating DXO.

In conclusion, we were able to simulate the physiological effects of DSM-TPCE with DXO in an *ex vivo* ILP model using human lung specimens. After DSM-TPCE, we did neither observe increased DXO concentrations in normal lung tissue nor histopathological signs of early lung toxicity. Therefore, the present results support further evaluation of DSM-TPCE in clinical trials.

## Data Availability

The datasets generated and/or analyzed during the current study are not publicly available due to continuous research on this topic but are available from the corresponding author upon reasonable request.

## Abbreviations

CT: Computed tomography
DSM: Degradable starch microspheres
DXO: Doxorubicin
ILP: Isolated lung perfusion
PAP: Pulmonary artery pressure
PVR: Pulmonary vascular resistance
STS: Soft tissue sarcoma
TLCr: Total lung capacity
TPCE: Transpulmonary chemoembolization
TTP: Time to peak
VATS: Video-assisted thoracoscopy
